# Rare acute myeloid leukemia harboring BCR/ABL1 (P210), AML1-MDS1/EVI1, and AML1/EAP: case report and literature review

**DOI:** 10.3389/fonc.2026.1917473

**Published:** 2026-08-20

**Authors:** Du Lijun, Fang Xu

**Affiliations:** Department of Hematology, Mianyang Central Hospital, Mianyang, Sichuan, China

**Keywords:** acute myeloid leukemia, BCR::ABL1, case report, EVI1 rearrangement, measurable residual disease

## Abstract

Acute myeloid leukemia (AML) with concurrent multiple fusion genes is extremely rare and characterized by high malignancy, poor chemotherapy response, and dismal prognosis. Here we report a 32-year-old male patient diagnosed with *de novo* high-risk AML harboring BCOR mutations, AML1-MDS1/EVI1, AML1-EAP, and BCR-ABL1 p210 fusion genes. The patient received induction chemotherapy with IA+VEN (idarubicin, cytarabine and venetoclax) regimen and achieved complete remission (CR); however, disease progression occurred during the first consolidation therapy. Subsequently, salvage chemotherapy combined with dasatinib was administered, and the patient reattained complete remission with incomplete hematologic recovery (CRi). The patient then underwent allogeneic hematopoietic stem cell transplantation (allo-HSCT) from his HLA-mismatched father. Post-transplant maintenance therapy consisting of chidamide, decitabine, and dasatinib was administered regularly. At the latest follow-up 3 months after transplantation, the patient remained in continuous molecular remission, with negative minimal residual disease (MRD) and significantly reduced BCR::ABL1 and EVI1 transcript levels. This case indicates that individualized sequential therapy including targeted agents, combination chemotherapy, allo-HSCT with chidamide-containing conditioning, and post-transplant epigenetic maintenance may provide effective disease control and favorable long-term outcomes for ultra-high-risk AML with multiple adverse molecular abnormalities.

## 1 Introduction

AML is a heterogeneous clonal hematologic malignancy derived from myeloid progenitor cells and characterized by impaired differentiation and uncontrolled proliferation, ultimately resulting in bone marrow failure. AML can occur at any age, with peak incidence observed in individuals aged 60–70 years (1). Molecular genetic abnormalities constitute the biological basis of AML heterogeneity and play critical roles in disease classification, risk stratification, prognostic assessment, and therapeutic decision-making (2).

According to the 2022 World Health Organization (WHO) classification of myeloid neoplasms, AML with BCR::ABL1 fusion and AML with MECOM rearrangement are categorized as high-risk subtypes. BCR::ABL1-positive AML accounts for approximately 0.5%–3% of AML cases, whereas MECOM-rearranged AML represents approximately 1%–2% (2). allo-HSCT remains the most important potentially curative treatment strategy for patients with high-risk AML (3). In addition, post-transplant maintenance therapy is increasingly recommended for high-risk patients and those with persistent measurable residual disease to reduce relapse risk (4).

Most published studies have focused on AML patients harboring a single fusion gene abnormality, whereas cases with multiple concurrent fusion genes are exceedingly rare. These patients often exhibit greater molecular complexity, marked clonal heterogeneity, and highly aggressive clinical behavior, posing substantial challenges for diagnosis and therapeutic management (5, 6). Herein, we report a young patient with ultra-high-risk AML harboring three concurrent fusion genes and a BCOR mutation, who was treated with sequential targeted therapy, chemotherapy, allo-HSCT, and post-transplant maintenance therapy. This case may provide additional clinical evidence and therapeutic insight into the management of AML with complex molecular abnormalities.

## 2 Patient information

The patient is a 32-year-old male, born on November 24, 1992.

## 3 Clinical presentation

The patient had a history of gout, for which he intermittently used uric acid-lowering medications and analgesics during acute attacks, although details of prior episodes and treatments were not fully documented. He had a smoking history of approximately 10 cigarettes per day and a prior alcohol consumption of approximately 100 ag per day, with no recent alcohol intake. No significant family history was reported.

The patient initially presented to our hospital on August 22, 2025, with persistent oral bleeding lasting for 9 days following tooth extraction. The bleeding was refractory to local hemostatic measures and antibiotic therapy. He denied associated systemic symptoms, including fever, dyspnea, or chest pain.

## 3 Diagnostic evaluation

### 3.1 Laboratory findings and imaging studies

Upon admission, complete blood count demonstrated leukocytosis (WBC 51.06 × 10^9/L), anemia (hemoglobin 109 g/L), and thrombocytopenia (platelet count 69 × 10^9/L). Differential counts revealed decreased lymphocytes (7.5%) and monocytes (1.7%), absent eosinophils (0%), slightly elevated basophils (3.3%), and circulating abnormal cells accounting for 22.5%. Coagulation studies showed prolonged prothrombin time (17.7 s), elevated international normalized ratio (INR 1.62), and reduced prothrombin activity (52.8%). Serum lactate dehydrogenase (718 U/L) and uric acid (786.8 μmol/L) levels were significantly increased.

Abdominal ultrasonography revealed mild hepatic steatosis, moderate splenomegaly, and an accessory spleen. Transthoracic echocardiography showed left atrial enlargement, focal aortic valve calcification, small pericardial effusion, and preserved left ventricular systolic function. Chest computed tomography revealed scattered small nodules in the right lung and left lower lobe suggestive of chronic inflammation, a small calcified focus in the right middle lobe, and patchy or nodular soft tissue density in the anterior mediastinum.

### 3.2 Bone marrow evaluation

Bone marrow aspiration performed on August 25, 2025, revealed a hypercellular marrow with 26.5% blasts. Myeloid cells accounted for 54%, while erythroid precursors were reduced to 7%, without evidence of dysplasia. Megakaryocytes were markedly decreased (n = 5), and platelets were scarce. Peroxidase (POX) staining showed 8% of the blasts were positive.

Flow cytometric immunophenotyping was performed on August 24, 2025. Analysis identified 19.4% abnormal myeloid blasts expressing CD34, CD117, CD123, CD33, HLA-DR, and CD13, with weak expression of CD38 and CD45. Partial expression was observed for CD11c (46.2%) and CD7 (54.3%).

Conventional karyotyping, reported on September 2, 2025, revealed 46,XY,t(9,22)(q34;q11)[4]/46,idem,t(3;21)(q26.3;q22)[16], indicating the presence of both the Philadelphia chromosome and an additional t(3;21) translocation.

Next-generation sequencing and fusion gene analysis were reported on September 2, 2025. NGS identified BCOR mutations (c.1024C>T, p.R342) and a frameshift mutation (c.1648_1651dup, p.T551Kfs*53). Fusion analysis detected AML1-MDS1/EVI1, AML1-EAP, and BCR::ABL1 p210. Quantitative PCR confirmed BCR::ABL1 positivity with an IS value of 123.25%.

HLA compatibility was assessed among family members. The patient’s father showed a GVH match of 10/12 and an HVG match of 9/12.

### 3.4 Treatment course

Upon admission, the patient received supportive care including plasma transfusion, hemostatic therapy, antimicrobial treatment, maintenance of oral hygiene, oral wound suturing, and hydroxyurea for cytoreduction.

**Figure 1:**
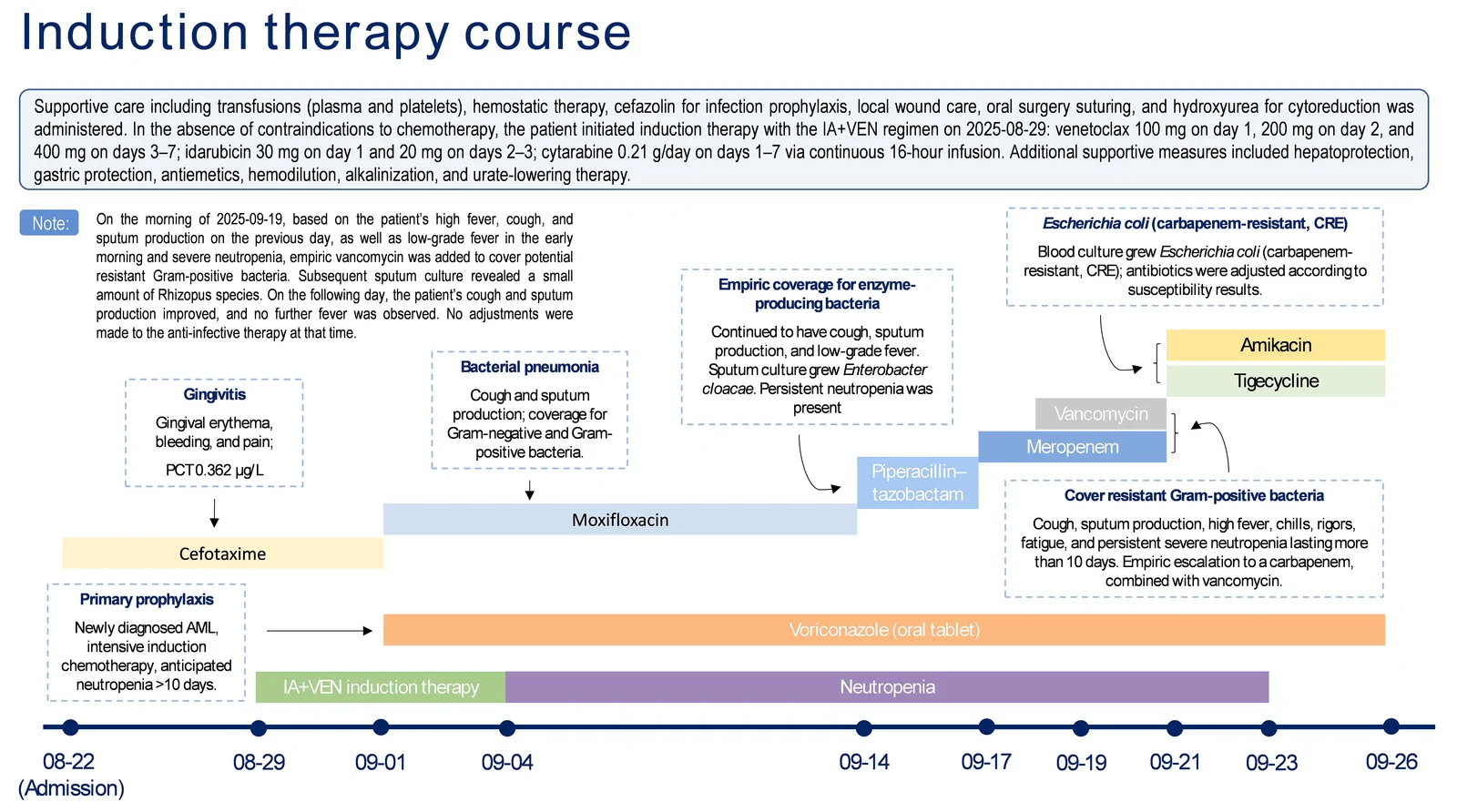
Clinical course during induction therapy. Blood culture grew Escherichia coli, which was positive for extended-spectrum β-lactamases (ESBL) and metallo-β-lactamases (MBL), but negative for serine-type carbapenemases. Antimicrobial susceptibility testing showed resistance to meropenem (MIC ≥16 μg/mL), piperacillin-tazobactam (MIC ≥128 μg/mL), and ceftazidime-avibactam (MIC 18 μg/mL), whereas susceptibility was observed for tigecycline (MIC ≤0.5 μg/mL) and amikacin (MIC ≤2 μg/mL).

Induction therapy with the IA+VEN regimen was initiated on August 29, 2025, consisting of venetoclax (100 mg on day 1, 200 mg on day 2, and 400 mg on days 3–7), idarubicin (30 mg on day 1 and 20 mg on days 2–3), and cytarabine (0.21 g/day on days 1–7 by continuous infusion). The patient achieved hematologic recovery after induction therapy. Bone marrow evaluation on day 21 showed no detectable blasts, and flow cytometry demonstrated MRD negativity (abnormal myeloid blasts <0.006%). However, quantitative PCR revealed persistent BCR::ABL1 p210 positivity, with a BCR::ABL1 p210/ABL(IS) ratio of 5.7083%. The induction course and subsequent treatment timeline are shown in **Figure 1**.

Given the achievement of morphologic remission with persistent molecular disease, consolidation therapy was initiated on October 10, 2025, consisting of cytarabine (2 ag intravenously every 12 hours on days 1–3) combined with dasatinib (100 mg orally once daily). Due to an episode of acute cholecystitis, bone marrow reassessment was postponed and performed on November 20, 2025. The evaluation showed hypercellular marrow with 7% blasts, and flow cytometry revealed 4.5% abnormal myeloid blasts among nucleated cells. Molecular assessment demonstrated persistent BCR::ABL1 p210 positivity (IS 91.39%) and EVI1 expression of 66.48%, while BCOR nonsense mutations were not detected. Cytogenetic analysis showed 46,XY,t(9;22) (q34;q11.2)[3]/46,XY[17].

The response to the first consolidation therapy was assessed as progressive disease (PD) due to delayed evaluation and persistent residual disease. Salvage therapy was initiated on November 21, 2025, with dasatinib (100 mg orally once daily) combined with the HA regimen (homoharringtonine 2 mg/day on days 1–7 and cytarabine 0.05 ag every 12 hours on days 1–7). Bone marrow evaluation on December 18, 2025, showed residual disease, with 1.4% abnormal myeloid blasts detected by flow cytometry. Molecular monitoring revealed persistent BCR::ABL1 p210 positivity (IS 27.58%) and EVI1 expression of 59.09%, while BCOR mutations remained undetectable. The response was evaluated as CRi with persistent flow cytometric and molecular MRD positivity.

Given the persistent molecular disease and ultra-high-risk genetic profile, the patient proceeded to allogeneic hematopoietic stem cell transplantation (allo-HSCT). The donor was the patient’s father with a 9/12 HLA match. Conditioning was performed using the ChiFAB regimen, followed by peripheral blood stem cell infusion on January 6, 2026. The patient achieved neutrophil and platelet engraftment on days +24 and +27, respectively. Bone marrow evaluation on day +27 demonstrated hematologic remission with negative flow cytometric MRD, BCR::ABL1 p210 positivity of 0.003%, low-level EVI1 expression, and donor chimerism >99%.

Considering the ultra-high-risk molecular features, post-transplant maintenance therapy was initiated with chidamide (5 mg twice weekly), dasatinib (100 mg daily), and decitabine (5 mg/m² on days 1–5 of each month). The patient remains under regular follow-up with serial hematologic and MRD monitoring.

### 3.5 Follow-up and outcomes

**Figure 2:**
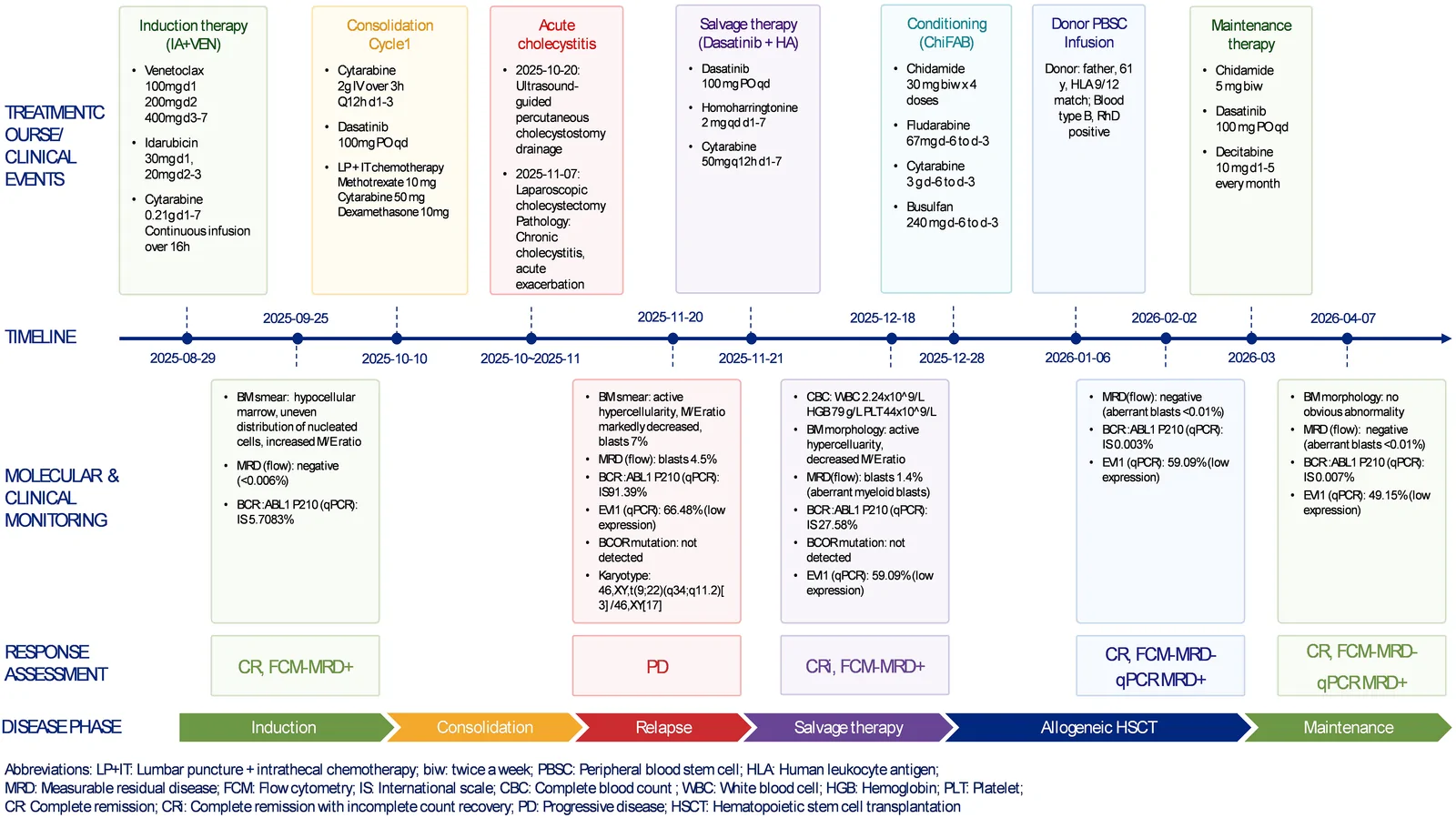
Timeline of the patient’s complete clinical course.

On April 7, 2026, a follow-up bone marrow evaluation was performed. Quantitative analysis of the EVI1 gene showed 49.15%, indicating low expression. Bone marrow flow cytometry revealed no abnormal phenotypic blast population, with minimal residual leukemic cells accounting for less than 0.01%. Quantification of BCR-ABL1 p210 showed an IS value of 0.007%. The patient is currently continuing maintenance therapy. An overview of the patient’s clinical course and treatment timeline is shown in **Figure 2**.

On July 13, 2026, follow-up bone marrow evaluation showed negative MRD by flow cytometry. Chromosomal karyotype analysis revealed a normal male karyotype (46,XY[20]). The EVI1 gene expression level was 3%, indicating low expression, and the BCR::ABL1 p210 transcript level was 2.676% on the IS. ABL1 kinase domain mutation analysis performed on a peripheral blood sample was negative. At the latest follow-up, the patient remained on maintenance therapy with dasatinib and decitabine.

## 4 Discussion

The present case represents an extremely rare form of acute myeloid leukemia (AML) harboring multiple adverse molecular abnormalities, including BCOR mutation, AML1-MDS1/EVI1 fusion, AML1-EAP fusion, and BCR-ABL1 p210 fusion. Such concurrent molecular lesions are exceptionally uncommon in *de novo* AML, and no standardized therapeutic strategy or established prognostic model currently exists for this subtype. A review of the PubMed-indexed literature identified no previously reported AML cases simultaneously harboring all three fusion genes observed in this patient. Although multiple fusion abnormalities have been described in other myeloid malignancies, particularly in chronic myeloid leukemia (CML) during accelerated or blast phases, reports of triple fusion gene-positive AML remain exceedingly scarce (7–10) (**Table 1**). Available evidence suggests that patients harboring two or more of these fusion genes are generally younger at onset, exhibit poor responses to chemotherapy, and frequently require allogeneic hematopoietic stem cell transplantation (allo-HSCT) to improve prognosis, consistent with the clinical features observed in our patient.

**Table 1:** Clinical characteristics of patients with hematologic malignancies harboring ≥2 of the three coexisting fusion genes in the index case, as reported in the literature.

Author	Year	Number of patients	Sex	Age (years)	AML1-MDS1/EVI1	AML1-EAP	BCR-ABLI	allo-HSCT	Outcome
Hamaguchi (1)	1997	1 (CMML transformed to AML)	Female	45	+	–	+	No	Deceased
Liu (2)	2006	1 (CML-BP)	Male	23	+	+	+	No	NR
Duan (3)	2023	1 *de novo* AML	Female	2.5	+	+		Yes	Alive
Alamri (4)	2024	1 *de novo* AML	Female	13	+	–	+	No	NR

allo-HSCT, allogeneic hematopoietic stem cell transplantation; CML-BP, chronic myeloid leukemia in blast phase; NR, not reported.

The AML1-MDS1/EVI1 fusion gene is generated by the t(3;21)(q26;q22) chromosomal translocation. It is extremely rare in *de novo* AML (<1%) and occurs more commonly in therapy-related MDS/AML or during progression of CML from chronic phase to blast crisis (11–13).

Previous retrospective studies have demonstrated that patients harboring t(3;21)(q26;q22) generally have highly aggressive disease, poor response to conventional chemotherapy, and extremely unfavorable outcomes (14, 15). In these studies, allo-HSCT appeared to be the only potentially effective strategy capable of achieving durable remission and prolonging survival. These findings further support the decision to pursue transplantation in the present case.

Previous studies demonstrated that the t(3;21)(q26;q22) translocation can generate multiple fusion transcripts, including AML1-MDS1/EVI1 and AML1-EAP. Both fusion products interfere with normal AML1-mediated transcriptional activation and hematopoietic differentiation. However, AML1-MDS1/EVI1 exhibits clear leukemogenic activity, whereas AML1-EAP appears to lack significant transforming capacity and may primarily function as a dominant-negative regulator of AML1 (16–18). Therefore, AML1-MDS1/EVI1 is considered the principal pathogenic driver in t(3;21)-associated myeloid malignancies, while AML1-EAP may serve as a cooperating molecular abnormality. In the present case, coexistence of AML1-MDS1/EVI1 and AML1-EAP may have contributed to increased molecular complexity and clonal heterogeneity.

High EVI1 expression has consistently been associated with adverse prognosis in AML. Groschel et al. demonstrated that elevated EVI1 expression correlated with lower complete remission rates, shorter event-free survival, and inferior overall survival (19). Consequently, high EVI1 expression is classified as a high-risk genetic abnormality in both the 2022 WHO classification and the European LeukemiaNet (ELN) risk stratification system (2, 20). In addition, several studies have shown that persistent or recurrent EVI1 positivity after allo-HSCT strongly predicts relapse and inferior leukemia-free survival (15, 21, 22). Therefore, dynamic monitoring of EVI1-related fusion transcripts may provide important prognostic information and facilitate early intervention in ultra-high-risk AML patients.

The BCR-ABL1 p210 fusion gene corresponds to the classical Philadelphia chromosome abnormality and occurs in only 0.5%–3% of adult AML cases (2). Historically, these patients exhibited poor outcomes, with median overall survival of approximately 7–9 months (6, 23). However, the introduction of tyrosine kinase inhibitors (TKIs) has significantly improved therapeutic outcomes in this subgroup (24, 25). Second-generation TKIs such as dasatinib appear to provide superior efficacy compared with first-generation TKIs because of their stronger inhibitory activity against BCR-ABL1 kinase and activity against most imatinib-resistant mutations (26, 27). Previous studies have shown that dasatinib-based regimens can achieve rapid hematologic and cytogenetic remission, particularly when combined with chemotherapy and allo-HSCT (28).

Importantly, coexistence of AML1-MDS1/EVI1 and BCR-ABL1 may exert synergistic leukemogenic effects. Cuenco GM et al. demonstrated in murine models that AML1-MDS1/EVI1 alone partially suppresses hematopoiesis but does not completely block myeloid differentiation. In contrast, co-expression of AML1-MDS1/EVI1 and BCR-ABL rapidly induces AML in murine models (29). This cooperative leukemogenic mechanism may partially explain the highly aggressive clinical course and poor response to conventional chemotherapy observed in the present patient.

In addition to fusion abnormalities, the coexisting BCOR mutation may further contribute to leukemogenesis. BCOR is an important regulator of epigenetic modification and cell-cycle control. Previous studies have shown that BCOR mutations may inhibit apoptosis, remodel the hematopoietic microenvironment, and promote clonal evolution (30). In the present case, BCOR mutation may have further enhanced disease aggressiveness and molecular instability.

An important differential diagnosis in this patient was CML in blast phase (CML-BP). However, several findings supported a diagnosis of *de novo* Philadelphia chromosome-positive AML rather than CML-BP, including the absence of a prior chronic-phase CML history, relatively short disease course, lack of marked basophilia (peripheral blood basophils accounted for only 3.3% at diagnosis), and the presence of multiple AML-associated molecular abnormalities. In addition, the patient primarily presented with acute leukemic manifestations rather than the typical chronic myeloproliferative features of CML. Morphologically, both peripheral blood and bone marrow examinations were predominantly composed of blasts, without the characteristic spectrum of granulocytic maturation, including increased myelocytes, metamyelocytes, band neutrophils, and segmented neutrophils, which is commonly retained in CML even during blast transformation. Although longitudinal monitoring of the Philadelphia chromosome or *BCR::ABL1* transcript was unavailable, the above clinical, hematologic, and molecular findings collectively favored a diagnosis of *de novo* AML rather than CML-BP. Post-transplant maintenance therapy may be particularly important in such ultra-high-risk AML patients. Previous studies have demonstrated that hypomethylating agents and histone deacetylase inhibitors, including decitabine and chidamide, may reduce relapse risk after allo-HSCT (4, 31–33). In addition, dasatinib maintenance may further suppress residual BCR-ABL1-positive leukemic clones. Therefore, combined maintenance strategies targeting both epigenetic dysregulation and BCR-ABL1 signaling may provide additional benefit in patients with complex molecular abnormalities.

In summary, this patient harbored four concurrent adverse molecular abnormalities, suggesting cooperative leukemogenic effects among multiple pathogenic pathways. These molecular features likely contributed to the highly aggressive disease biology and poor response to conventional chemotherapy. This case highlights the importance of comprehensive molecular profiling in AML patients with atypical clinical features and suggests that early incorporation of TKI-based therapy and allo-HSCT may be critical for achieving durable remission in such ultra-high-risk molecular subtypes.

Nevertheless, several limitations of this case should be acknowledged. The current follow-up period remains relatively short, and therefore the long-term clinical outcome and durability of remission cannot yet be determined. The patient is currently undergoing continuous follow-up with serial MRD monitoring every three months. Continued surveillance will provide further information regarding the long-term efficacy of the current treatment strategy, the risk of relapse, and post-transplant disease control. If appropriate, we intend to report updated clinical outcomes in future follow-up publications.

## Data Availability

The original contributions presented in the study are included in the article/supplementary material. Further inquiries can be directed to the corresponding author.
